# Portable Ultrasound-Based Device for Detecting Older Adults’ Sit-to-Stand Transitions in Unsupervised 30-Second Chair–Stand Tests

**DOI:** 10.3390/s20071975

**Published:** 2020-04-01

**Authors:** Antonio Cobo, Elena Villalba-Mora, Dieter Hayn, Xavier Ferre, Rodrigo Pérez-Rodríguez, Alberto Sánchez-Sánchez, Raquel Bernabé-Espiga, Juan-Luis Sánchez-Sánchez, Andrea López-Diez-Picazo, Cristian Moral, Leocadio Rodriguez-Mañas

**Affiliations:** 1 Centre for Biomedical Technology (CTB), Universidad Politécnica de Madrid (UPM), Pozuelo de Alarcón, 28223 Madrid, Spain; antonio.cobo@ctb.upm.es (A.C.); xavier.ferre@ctb.upm.es (X.F.); alberto.sanchez@ctb.upm.es (A.S.-S.); cristian.moral@ctb.upm.es (C.M.); 2 Centro de Investigación Biomédica en Red en Bioingeniería, Biomateriales y Nanomedicina (CIBER-BBN), Madrid, 28029 Madrid, Spain; 3 Digital Health Information Systems, Austrian Institute of Technology (AIT), Graz, 8020 Styria, Austria; dieter.hayn@ait.ac.at; 4 Fundación para la Investigación Biomédica, Hospital de Getafe, Getafe, 28905 Madrid, Spain; rprodrigo@salud.madrid.org (R.P.-R.); raquel.bernabe@salud.madrid.org (R.B.-E.); juanluis.sanchez@salud.madrid.org (J.-L.S.-S.); andreajulia.lopez@salud.madrid.org (A.L.-D.-P.); 5 Servicio de Geriatría, Hospital de Getafe, Getafe, 28905 Madrid, Spain; leocadio.rodriguez@salud.madrid.org; 6 Centro de Investigación Biomédica en Red en Fragilidad y Envejecimiento Saludable (CIBER-FES), 28029 Madrid, Spain

**Keywords:** frailty syndrome, sit-to-stand, 30-s chair stand test, ultrasound, signal processing

## Abstract

Lower-limb strength is a marker of functional decline in elders. This work studies the feasibility of using the quasi-periodic nature of the distance between a subjects’ back and the chair backrest during a 30-s chair–stand test (CST) to carry out unsupervised measurements based on readings from a low-cost ultrasound sensor. The device comprises an ultrasound sensor, an Arduino UNO board, and a Bluetooth module. Sit-to-stand transitions are identified by filtering the signal with a moving minimum filter and comparing the output to an adaptive threshold. An inter-rater reliability (IRR) study was carried out to validate the device ability to count the same number of valid transitions as the gold-standard manual count. A group of elders (age: mean (m) = 80.79 years old, SD = 5.38; gender: 21 female and seven male) were asked to perform a 30-s CST using the device while a trained nurse manually counted valid transitions. Ultimately, a moving minimum filter was necessary to cancel the effect of outliers, likely produced because older people tend to produce more motion artefacts and, thus, noisier signals. While the intra-class correlation coefficient (ICC) for this study was good (ICC = 0.86, 95% confidence interval (CI) = 0.73, 0.93), it is not yet clear whether the results are sufficient to support clinical decision-making.

## 1. Introduction

**Figure 1:**
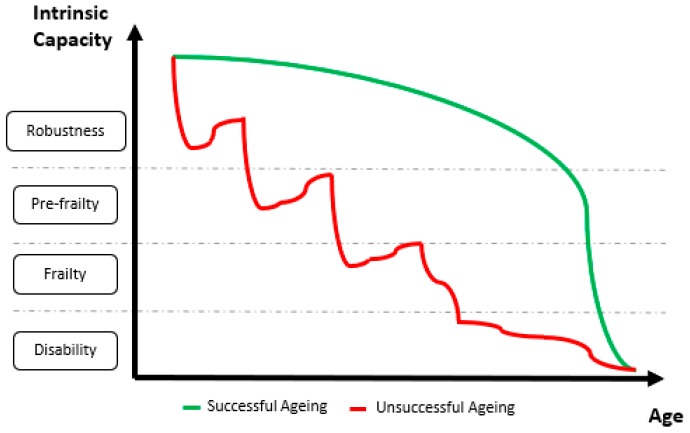
Graphical representation of simulated successful and unsuccessful aging trajectories. The horizontal axis represents the advance of time from the beginning of the old age to the end of life. The vertical axis represents the categories in which patients fall along the progress of functional decline (from robustness to disability). The green line represents a successful aging trajectory: good functional status is enjoyed for most of the old age and functional decline only happens rapidly and close to the end of life. The red line represents an unsuccessful aging trajectory: a clear trend of fast functional decline is observed throughout old age together with a long period of disability, several years long, before the end of life.

The purpose of the present work is to develop a portable and autonomous device for older people to self-administer the 30-s chair–stand test (CST) at their homes. It is meant to be used by patients with the help of informal caregivers, to measure lower-limb strength according to their geriatrician’s established plan, (i.e., once or twice a week). The 30-s CST is a physical performance assessment tool; in particular, it measures lower-limb strength [1]. It does so by quantifying the ability of older patients to repeatedly stand up from a chair, by specifically counting the number of times they stand up over thirty seconds [1]. Poor physical performance in older patients is strongly related to undesired clinical outcomes such as disability, hospitalization, deaths [2], and falls [3]. Disability and all these related outcomes are the culmination of a progressive process of physiological decline [4]. A successful aging path will delay the onset of disability as late in life as possible (green line in Figure 1) as opposed to an unsuccessful aging path that will involve being disabled for several years (red line in Figure 1) [5].

Within the context of an aging population [6], the social and the economic impacts of delaying the onset of dependency would be enormous because poor functionality has a strong impact on patients’ and relatives’ quality of life and makes them big users of health and social services [7]. However, despite the remarkable increase in longevity enabled by healthcare systems, they do not achieve an extension of the period of good health and functionality [7]. Fortunately, it is indeed possible to extend good health and functionality because, even though disability is not reversible, it is preceded by a reversible stage known as frailty [8,9,10]. By detecting the onset of frailty at early stages, frail people can be restored back to a robust status thanks to exercise-based interventions [8,9,10].

Several models were proposed to explain frailty, which is defined as a state of increased vulnerability to adverse outcomes due to a reduction in the ability to respond to stressors [4,11]. Of course, all of these models identify physical performance as a strong frailty marker [4,12]. In particular, lower-limb strength is one of the criteria for patient frailty stratification according to the frailty phenotypic model [4]. There are multiple clinical tests to assess lower-limb strength; the most remarkable of them are based on assessing performance under repeated sit-to-stand conditions. The Short Physical Performance Battery (SPPB), for instance, is based on recording how long it takes for an older person to repeatedly stand up from a chair five times [13]. Conversely, the 30-s CST is based on recording how many times a person stands up from a chair over 30 s [1]. Any of them can be found in comprehensive geriatric assessment (CGA) tools, which are proposed as the most valuable tools to inquire the advance of frailty, to predict and prevent sudden adverse events, and to develop intervention plans for the delay and reversal of the onset of frailty [14,15,16].

An automated version of the 30-s CST is expected to count the same number of completed cycles as a manual count would do. It requires the automatic identification and delimitation of sit–stand–sit (STS) cycles and the ability to automatically spot and dismiss failed attempts (i.e., when the subject does not reach an upright posture). Inertial sensors and inertial measurement units (IMUs) were extensively used to study sit-to-stand and stand-to-sit transitions, as well as STS cycles, over the past three decades [17]. Some of these studies looked for relationships between different kinematic parameters and the functional status (robust, pre-frail, frail) of the experimental subjects [18,19,20]. A very popular approach is based on taking advantage of the quasi-periodic nature of the trunk movement during STS cycles [21,22]. To do so, previous works used signals from an IMU on the subject’s lower back [21,22].

Van Lummel et al. used triaxial acceleration and triaxial angular velocity signals (dynaport device) to compute trunk pitch angle and vertical velocity signals [21], while Millor et al. used the vertical acceleration signal (MTx Orientation Tracker–Xsens Technologies B.V. Enschede, the Netherlands) to compute vertical velocity and vertical position [22]. Computing velocity from an acceleration signal requires the integration of the acceleration signal. Modern accelerometers are very small but noisy micro-electro-mechanical systems (MEMS); therefore, the integration of their noisy output results in a drifting velocity signal. They require a heavy pre-processing stage to compensate for the said drift. In particular, Millor et al. reported that, on the one hand, they applied double integration combined with fourth-level polynomial curve adjustment and cubic spline interpolation; on the other hand, they relied on the MTx onboard Kalman filter estimation for the X-orientation to identify the STS sub-phases in combination with the vertical acceleration, the vertical velocity, and the vertical position [22].

The wearable devices described above require the IMUs to be placed on the L3 region of the subject’s lumbar spine [17,22]. Older people might experience some difficulties in placing them on the correct spot, especially if they do not have any help to put them on. These previous studies did not study their algorithm sensitivity to misplacing the sensor; therefore, further studies are necessary to test the usability of these kinds of systems for an older population, as well as to assess the elders’ overall user experience, especially outside controlled experimental settings.

We studied the feasibility of using the quasi-periodic nature of the distance between a subject’s back and the chair backrest during a 30-s CST to carry out unsupervised measurements based on readings from a low-cost ultrasound sensor. Our search in PubMed for studies based on ultrasound sensors did not return any eligible results. We observed the feasibility of such an approach to vary depending on the age and/or functional status of the user. The target population for this kind of clinical test involves older adults. Unfortunately, we observed them to generate such noisy signals that local maxima became hard to match actual sit-to-stand transitions. Using a moving minimum filter to cancel the effects of said noise resulted in a remarkable improvement. Nevertheless, it is still not clear whether the results are good enough to make any clinical decisions based on the sensor outcomes.

## 2. Materials and Methods

A purely observational study was carried out to collect digital distance signals. These signals were used to develop and validate an algorithm able to spot and count sit-to-stand transitions. The algorithm was developed in an iterative process. A preliminary version of the algorithm was based on signals from a group of healthy subjects. A group of older subjects provided a second set of signals, which were used to refine the algorithm to its latest version.

### 2.1. Participants

Two different groups of subjects were involved, namely, healthy young subjects and older subjects. All the subjects gave their informed consent for inclusion before they participated in the study. The study was conducted in accordance with the Declaration of Helsinki, and the protocol was approved by the Ethics Committee for Clinical Research of the University Hospital of Getafe on 12 January 2018 with protocol code “FACET, versión v3”.

#### 2.1.1. Healthy Young Subjects (Group A)

Each subject in group A performed a 30-s CST test in a controlled laboratory environment. The resulting signals were recorded and stored as dataset A. This dataset was used to develop Algorithm-v1.

#### 2.1.2. Older Subjects (Group B)

Thirty older subjects were recruited from a pool of participants that expressed a general interest in participating in research studies from the University Hospital of Getafe (HUG). Patients were contacted from this pool by phone and asked whether they were willing to respond to some screening questions. Inclusion and exclusion criteria can be found in Table 1. Gender and age information from two participants were missing; thus, the following demographic data are reported based on data from the 28 remaining participants (age: mean (m) = 80.79 years old, SD = 5.38; gender: 21 female and seven male).

Each subject in group B performed a 30-s CST in a pre-clinical environment at the HUG venues. The resulting signals were recorded and stored as dataset B. This dataset was used to validate Algorithm-v1, and to develop and validate Algorithm-v2.

### 2.2. Apparatus

**Figure 2:**
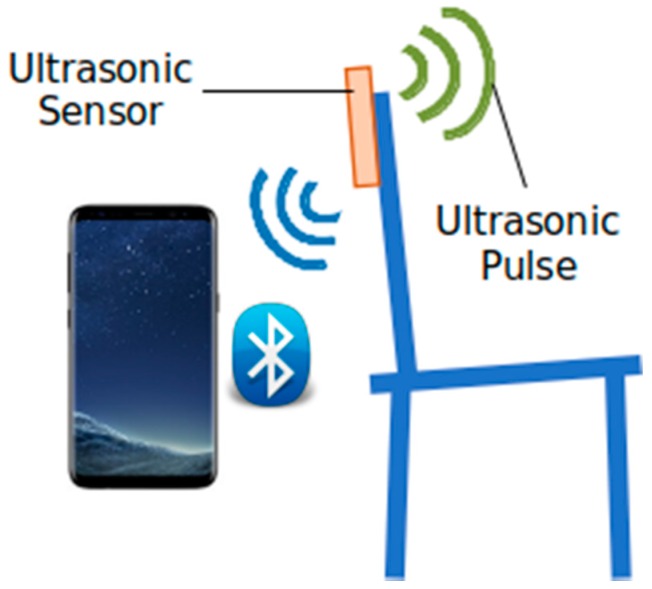
Depiction of the experimental set-up. The chair–stand sensor was attached to the backrest of a regular rigid chair. The chair–stand sensor emitted ultrasonic pulses toward the subject’s back. An Android app in a mobile device was used to control the chair–stand sensor via Bluetooth and to store the sensor readings for subsequent analysis.

The overall set-up consisted of an electronic device equipped with a low-cost ultrasound sensor, hereon chair–stand sensor, a chair, and a tablet device. The chair had to be a regular rigid chair with a backrest. The chair–stand sensor was attached to the chair backrest and communicated with the tablet device via Bluetooth as shown in Figure 2. The members of the research team used an ad hoc app in the tablet device to configure the sensor, to start a 30-s CST, and to read the sensor outcomes at the end of each test.

#### 2.2.1. The Chair–Stand Sensor

**Scheme 1:**
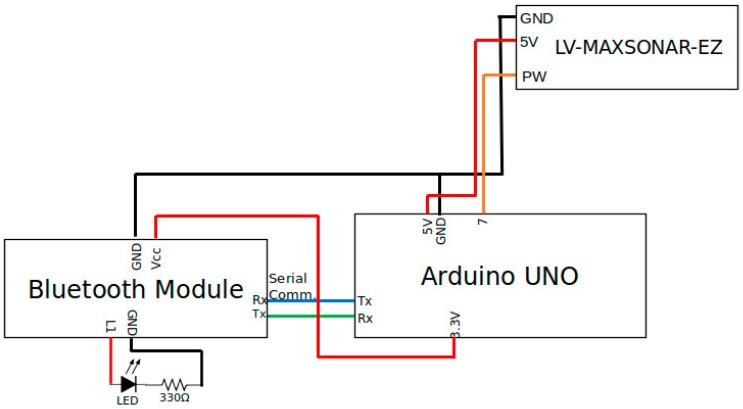
Schematic diagram of the chair–stand sensor. An Arduino UNO board was in control of the ultrasound sensor and collected its distance readings. It made use of the Bluetooth module to exchange messages with the external mobile device. The Arduino UNO board was powered by six AA batteries and was used to power the other two modules. The batteries are omitted in this scheme for the sake of clarity.

The chair–stand sensor consisted of three main building blocks as shown in Scheme 1. These building blocks were an Arduino UNO board, a MaxBotix LV-MAXSONAR-EZ ultrasound sensor, and a Bluetooth 2.0 + EDR module (HC-06). Additionally, the device included a little case for six AA batteries as power source. The batteries were omitted in Scheme 1 for the sake of clarity.

The Arduino board acted as the processing unit in the device thanks to its onboard micro-controller. It governed the behavior of the ultrasound sensor and made use of the Bluetooth module to exchange messages with the tablet device. The ultrasound sensor was powered by the Arduino board itself. The Arduino board had an ultrasound sensor to emit a pulse every 100 ms, and it read and recorded the corresponding echo values. The effect as a distance signal was sampled at a 10-Hz rate.

The Bluetooth module was also powered by the Arduino board and exchanged messages with the Arduino board via serial communications (blue and green wires in Scheme 1). The Bluetooth module controlled a light-emitting diode (LED) as an indicator of operational status.

**Figure 3:**
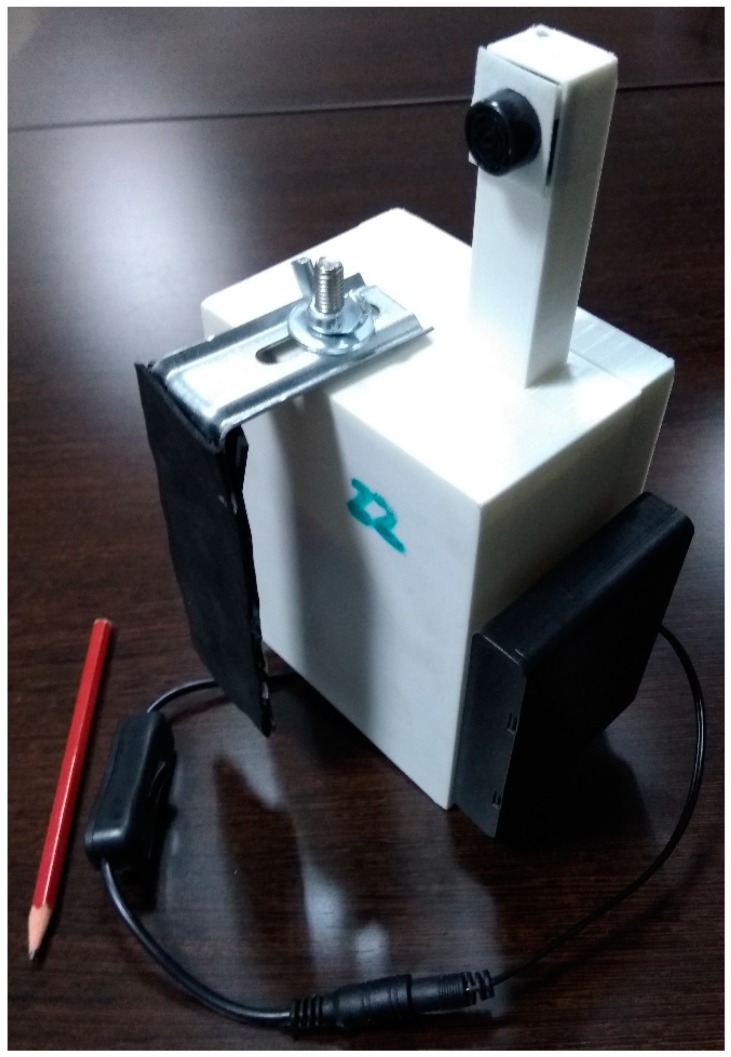
External casing for the chair–stand sensor. The sensor can be attached to chairs with very different designs thanks to a hook of adjustable width. The ultrasound sensor towers above like a periscope.

The whole system was protected by a resistant casing to ensure the integrity of the device, as shown in Figure 3.

#### 2.2.2. The Tablet App

The application was developed in Java for Android. The tablet device was a Huawei M2-A01L with Android 5.1.1.

#### 2.2.3. The Algorithms

Two algorithms were developed. Both of them spot sit-to-stand transitions by detecting local maxima and minima in the digital distance signal. The algorithms were developed taking advantage of the fact that the distance is expected to vary in a predictable way, resulting in a semi-periodic signal. Both algorithms comprised three stages: preprocessing, peak detection, and decision. They were developed in GNU Octave version 4.4.1 and required the use of the signal and nan packages. The code is available as Supplementary Material.

In the preliminary algorithm, hereon Algorithm-v1, the preprocessing stage was used to detect and reset outliers. All sample values over 70 cm (which is way over the maximum expected distance for an upright position) were set to 15 cm (an estimation of the usual distance for a sitting position). A moving median filter (window length of 0.7 s) was applied to the preprocessed signal. Local maxima in the filtered signal were considered to be a single peak if they were separated by less than a given threshold known as the peak distance threshold (PDT). The resulting peaks were considered to be successful sit-to-stand transitions if they exceeded another given threshold known as the peak height threshold (PHT). The PDT was computed as the median distance between peaks in the filtered signal multiplied by a given factor known as the distance factor (DF). Ten signals from dataset A (healthy subjects) were used to adjust the value of DF and PHT by minimizing the mean error between the outcomes of the algorithms and manual counts.

In the second algorithm, hereon Algorithm-v2, the threshold value for outlier detection in the preprocessing stage was increased from 70 to 99 cm. All sample values over 99 cm were set to NaN (Octave’s label for non-defined values). A moving minimum filter (window length 0.7 s) was applied to the preprocessed signal. An adaptable threshold known as the sitting–standing threshold (SST) was applied to the filtered signal to obtain a binary signal with two possible states, i.e., if the subject is sitting or standing. The time points when the subject stood up were detected by spotting changes in the sign of the binary signal’s derivative. All standing events closer to each other than a given threshold known as the minimum peak distance (MPD) were merged together into a peak. The MPD was measured in seconds. The resulting peaks were considered eligible candidates for sit-to-stand transitions and were further processed to merge peaks closer to each other than a given threshold known as the minimum samples between peaks (MSBP) and to discard peaks smaller than another given threshold known as the minimum subject distance (MSD). The MSBP was measured as the number of samples and the MSD was measured in cm. The adaptive threshold SST was defined as the sum of the moving minimum plus the weighed subtraction of the moving minimum from the moving median of the preprocessed signal (using a 4-s window for both moving filters). The weight is known as the adaptive threshold weight (ATW). Ten signals form dataset B (older subjects) were used to adjust the values of the ATW, the MPD, the MSBP, and the MSD.

### 2.3. Procedure

#### 2.3.1. Collecting Dataset A

Twenty-five healthy young subjects were each administered a 30-s CST. Subjects were asked to repeatedly stand up from and sit down on a chair for 30 s. The chair was equipped with the chair–stand sensor described before. The digital distance signals were recorded for further processing. A trained human rater counted sit-to-stand transitions and labeled them as non-valid if the subject did not reach an upright posture.

#### 2.3.2. Collecting Dataset B

Thirty older subjects participated in the study. The total duration of participation for each subject was restricted to one visit with no follow-up. At the same visit, the SPPB, the Linda Fried’s Criteria for Frailty, and the 30-s CST were administered. The following demographic information was also collected: date of birth, gender, education level (years of school and higher education), and primary occupation.

As for the 30-s CST, subjects were asked to repeatedly stand up from and sit down on a chair for 30 s. The chair was equipped with the chair–stand sensor described before. The digital distance signals were recorded for further processing. A trained nurse counted sit-to-stand transitions and labeled them as non-valid if the subject did not reach an upright posture.

### 2.4. Analysis

#### 2.4.1. Counting Sit-to-Stand Transitions

The 30-s CST is usually administered in a clinical context by a trained health professional. We needed our device to assign test scores like a trained professional would do. For that reason, we treated our scenario as an inter-rater reliability problem.

Not every sit-to-stand event in a 30-s CST is valid. Sit-to-stand events must not add to the overall count when the subject stands up from the chair but does not reach a complete upright position. In order to test the impact of invalid sit-to-stand events on the algorithm performance, we conducted separated IRR tests for valid sit-to-stand events and total sit-to-stand events.

**IRR tests for Algorithm-v1**

Three IRR tests were conducted:

1. Between manual count and Algorithm-v1 on dataset A.
2. Between manual count (valid events) and Algorithm-v1 on dataset B.
3. Between manual count (total events) and Algorithm-v1 on dataset B.

**IRR tests for Algorithm-v2**

Two IRR tests were conducted:

4. Between manual count (valid events) and Algorithm-v2 on dataset B.
5. Between manual count (total events) and Algorithm-v2 on dataset B.

In all five tests, IRR was assessed by means of computing the intra-class correlation coefficient (ICC). ICC estimates and their 95% confidence intervals (CI) were calculated using the *irr* package in the R statistical software, version 3.6.2. IRR tests involved each a single-rater, absolute-agreement, two-way model. As recommended by Koo and Li (2016, p. 155), “based on the 95% confident interval of the ICC estimate, values less than 0.5, between 0.5 and 0.75, between 0.75 and 0.9, and greater than 0.90 are indicative of poor, moderate, good, and excellent reliability, respectively”.

#### 2.4.2. Classifier Performance

Rikli and Jones identified the normative standards to use the 30-s CST scores to classify patients as (i) within, (ii) below, or (iii) over the reference performance range of the average population [23]. The expected level of physical decline due to normal aging varies depending on gender and age. Thus an 82-year-old woman with a score equal to 11 would be in the average class while a 63-year-old woman with the same score would be in the below-average class. Analogously, a 62-year-old woman with a score equal to 13 would be in the average class, while a man with the same age and score would be in the below-average class.

We used Rikli and Jones’s tables of normative data [23] to translate the manual counts and the Algorithm-v2 scores of each subject in dataset B (older adults) into one of three classes: (i) average (between the 25th and 75th percentiles), (ii) below average (below the 25th percentile), and (iii) above average (above the 75th percentile) [23]. Then, we tested the classifier performance. Such a classifier would be useful if it were able to provide an accuracy greater than always assigning the average class (which is the most common class). In dataset B, always assigning the average class provides an accuracy of 75% but it does not provide any useful information about the subject’s functional status; therefore, we refer to it as the no information rate (NIR).

Accuracy was obtained together with its 95% CI. Accuracy was computed as the overall number of correct classifications divided by the total number of tests. A single-sided binomial test was conducted to test whether accuracy was greater than the NIR (as an alternative hypothesis). All these parameters were calculated using the confusionMatrix function from the caret package in the R statistical software, version 3.6.2. Effect size and power value were calculated, respectively, with the ES.h function and the pwr.p.test from the pwr package in the R statistical software, version 3.6.2.

## 3. Results

### 3.1. Sit-to-Stand Event Count

Table 2 shows the results of the IRR study for Algorithm-v1.

IRR between the human rater and Algorithm-v1 was excellent (ICC > 0.9) when applied to dataset A (healthy subjects). This observation was supported by all three parameters: (i) the coefficient estimate (ICC = 0.96), (ii) the 95% CI, and (iii) the significance value. This result suggests that Algorithm-v1 is equivalent to human assessment.

However, the results of Algorithm-v1 applied to dataset B (older subjects) showed poor equivalency to human assessment. When comparing the outcomes from Algorithm-v1 to the manual count of valid events, the ICC value (ICC = 0.50) showed moderate levels of correlation. However, statistical significance values were not even able to ensure ICC levels over 0.5. This result suggest that Algorithm-v1 cannot be used to identify valid sit-to-stand events from the ultrasound-based distance signal on an elderly population.

The same happened when Algorithm-v1 outcomes were compared to the manual count of total events. The ICC value (ICC = 0.50) improved a little but still showed moderate levels of correlation. Again, statistical significance values were not even able to ensure ICC levels over 0.5. Thus, correlation between the outcomes cannot be considered better than poor. This result suggest that Algorithm-v1 cannot be used to detect the total number of sit-to-stand events, regardless of them being valid or invalid, from the ultrasound-based distance signal on an elderly population.

**Figure 4:**
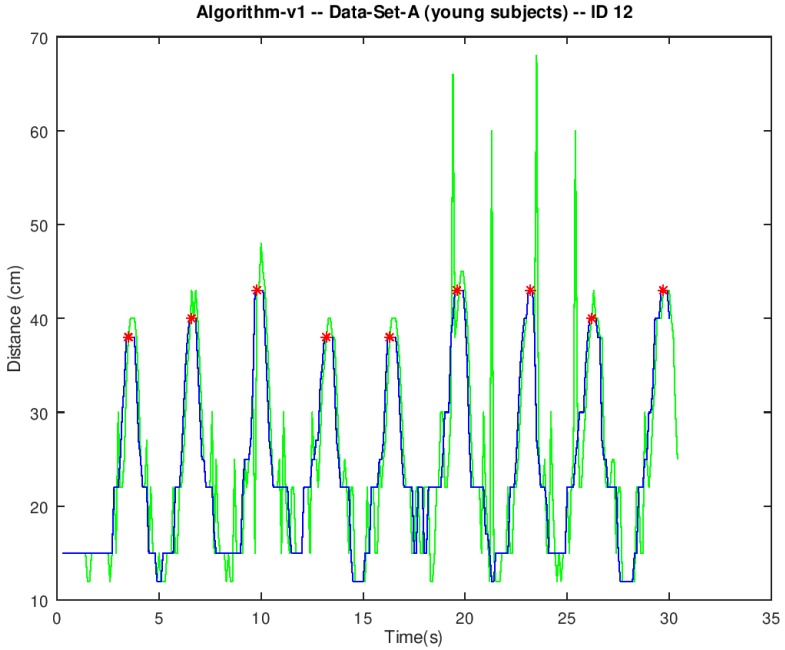
Graphical representation of a signal from dataset A (young healthy subjects) and the outcomes resulting from processing it with Algorithm-v1. The green line represents the pre-processed signal after removing all the outliers over a given threshold. The blue line represents the output of applying a moving median filter to the green signal. The red dots represent the sit-to-stand transitions identified and reported by the algorithm.

We observed the signals in dataset A to show a few spurious spikes even after removing outliers in the pre-processing stage (green line in Figure 4). These spikes are indeed outliers that fell below the threshold in the pre-processing stage. We observed legitimate peaks up to 50 cm to be rather common within the datasets. Nevertheless, lowering the pre-processing threshold to 50 cm did not show any significant improvement. Therefore, we decided to keep the pre-processing threshold high not to make any legitimate peaks unrecognizable. Spurious spikes in dataset A were narrow, spaced wide apart, and scarce. Therefore, they did not pass through the moving median filter (blue line in Figure 4). Even though they broke the smooth progression of the curve, they did not affect the overall shape and the quasi-periodic nature of the signal. Therefore, all peaks were correctly detected (red dots in Figure 4).

**Figure 5:**
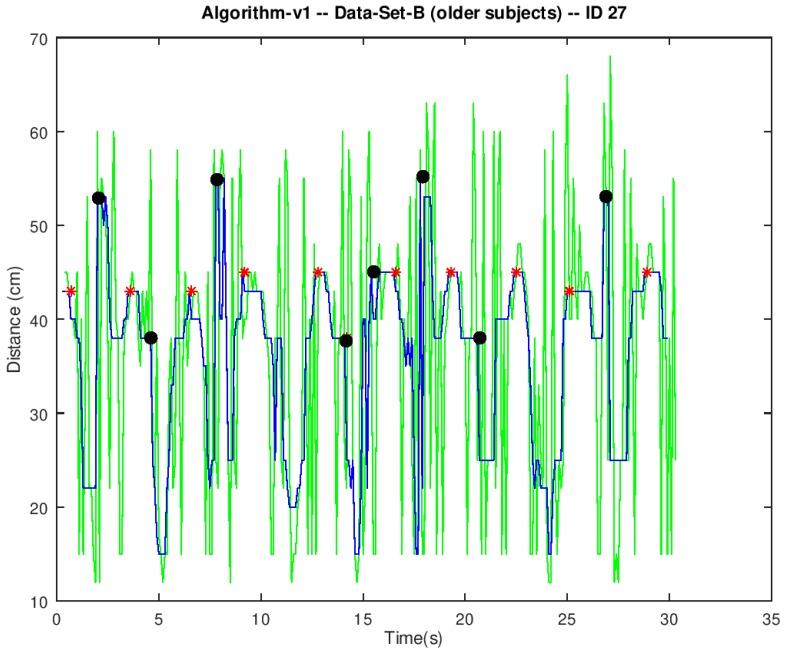
Graphical representation of a signal from dataset B (older subjects) and the outcomes resulting from processing it with Algorithm-v1. The green line represents the pre-processed signal after removing all the outliers over a given threshold. The blue line represents the output of applying a moving median filter to the green signal. The red dots represent legitimate sit-to-stand transitions identified and reported by the algorithm. The black dots represent sit-to-stand transitions erroneously identified and reported due to the effect of the spurious spikes in the green signal.

Conversely, spurious spikes in dataset B were abundant and very close to each other (green line in Figure 5). Therefore, their contribution to the output of the moving median filter (blue line in Figure 5) could result in such a significant distortion that the peak detection algorithm mistook it for a legitimate peak (black dots in Figure 5).

Table 3 shows the results of the IRR study for Algorithm-v2.

Algorithm-v2 was directly applied to dataset B (older subjects). When comparing the outcomes from Algorithm-v2 to the manual count of valid events, the ICC value (ICC = 0.86) showed good levels of correlation. This observation was corroborated by the fact that statistical significance values were able to ensure ICC levels over 0.75. This result is a remarkable improvement over Algorithm-v1. In fact, the 95% CI suggests that the true ICC value could very well fall within the excellent correlation range; the question remains whether these levels of reliability are good enough to make any clinical decisions based on outcomes from the chair–stand sensor.

When the outcomes from Algorithm-v2 were compared to the manual count of total events, the ICC value (ICC = 0.89) improved a little. It remained within the range of good levels of correlation, but it could not be considered excellent. Again, statistical significance values supported that the true ICC value was over 0.75. This result suggests that the presence of invalid sit-to-stand events had very little impact on Algorithm-v2 performance. Thus, the mistakes preventing Algorithm-v2 from showing an excellent correlation with manual count did not seem to come from a systematic erroneous classification of invalid sit-to-stand transitions. Moreover, the data showed neither systematic overestimation nor underestimation of sit-to-stand transitions. Nine of the signals resulted in an overestimated count and another nine signals resulted in an underestimated count. Thus, the noise present in the distance signal seemed to be equally likely to either mask or simulate valid sit-to-stand transitions.

**Figure 6:**
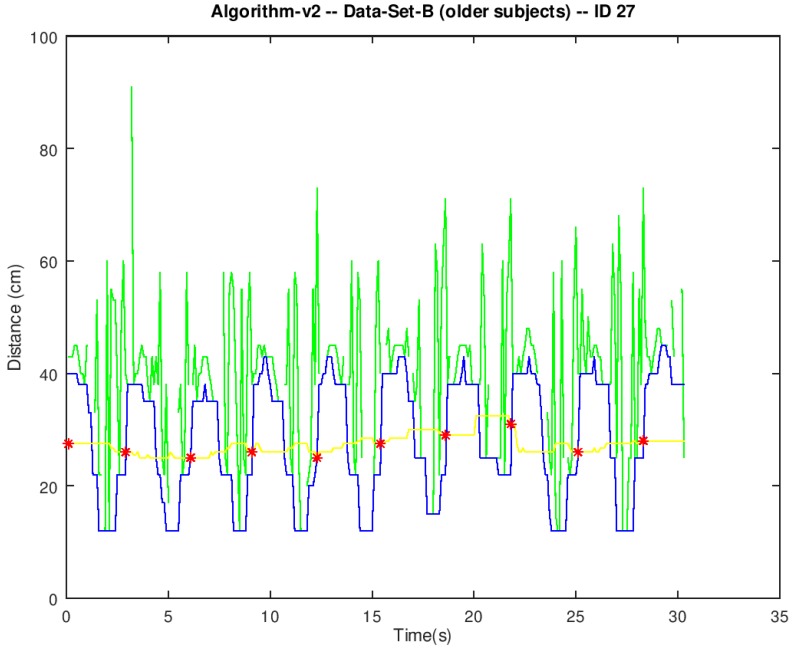
Graphical representation of a signal from dataset B (older subjects) and the outcomes resulting from processing it with Algorithm-v2. The green line represents the pre-processed signal after removing all the outliers over a given threshold. The blue line represents the output of applying a moving minimum filter to the green signal. The filter completely cancels the effect of the spurious spikes in the green signal. The red dots represent the sit-to-stand transitions identified and reported by the algorithm.

Algorithm-v2 applies a moving minimum filter to the pre-processed signal instead of the moving median filter in Algorithm-v1. The output of the moving minimum filter (blue line in Figure 6) followed the envelope of the local minima in the pre-processed signal (green line in Figure 6). Legitimate sit-to-stand events (red dots in Figure 6) were easier to spot on the envelope of the local minima because such a filtered signal was freed from the rapid variations of the spurious spikes.

**Figure 7:**
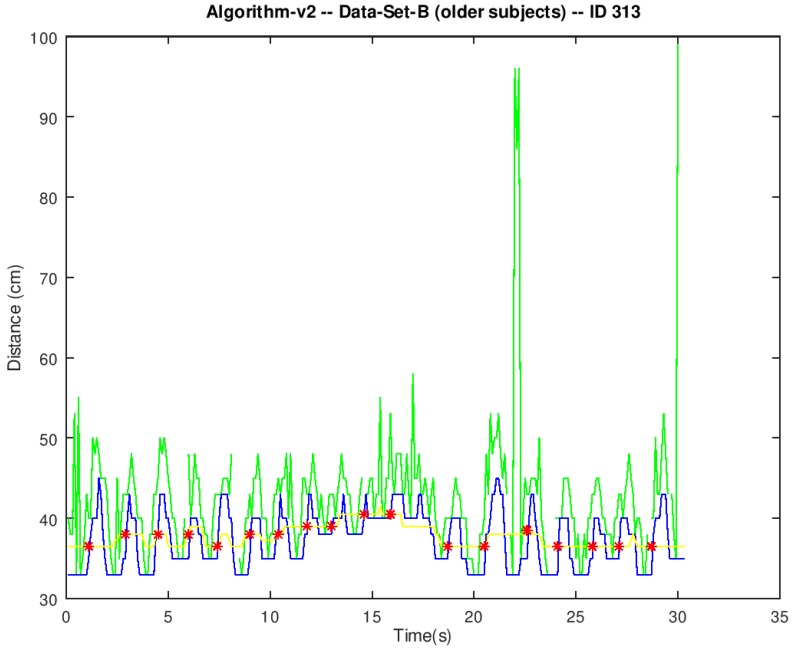
Graphical representation of a signal from dataset B (older subjects) and the outcomes resulting from processing it with Algorithm-v2. The green line represents the pre-processed signal after removing all the outliers over a given threshold. The blue line represents the output of applying a moving minimum filter to the green signal. The red dots represent the sit-to-stand transitions identified and reported by the algorithm. Even though the filter canceled the adverse effects from the spurious spikes, the algorithm missed one sit-to-stand transition. Like many other examples of incorrect count, the filtered signal presented minima with values over 30 cm.

Even though Algorithm-v2 canceled the effect of the spurious spikes, it was still not fully correlated to manual count. We observed that the algorithm had difficulties in correctly spotting sit-to-stand transitions when the value of the minima in the filtered signal (blue line in Figure 7) was greater than 30 cm. Most of them missed the correct result by one event. The example in Figure 7 reported one fewer sit-to-stand event than the manual count. Presumably, the algorithm failed to spot the peak around t = 17 s. Other examples with minima greater than 30 cm reported more events than manual counts. It is hard to tell which were the erroneous peaks from visual inspection.

### 3.2. Classifier Performance

Data from two participants in dataset B were missing information about their gender and age. Since this information is essential to translate the 30-s CST numerical scores into classes of functional performance (i.e., average, below average, and above average), we had to remove their scores, and we did not to use them to assess the classifier performance.

Table 4 shows the results of the classifier performance assessment.

The results of the classifier performance assessment were not conclusive. The estimate for the classifier accuracy was greater than the NIR. However, the binomial test was not significant; thus, we could not guarantee that the actual accuracy of the classifier was really greater than the NIR. The 95% CI was consistent with this observation. In any case, the low power of the analysis did not allow us to firmly state that the accuracy was not greater than the NIR either.

## 4. Discussion

The excellent IRR results obtained for Algorithm-v1 on dataset A (young healthy subjects) were very promising. However, a subsequent IRR analysis showed Algorithm-v1 to be useless when applied to dataset B (older subjects). The subset of signals in dataset A used in adjusting Algorithm-v1 parameters were also used in performance testing. Thus, an overfitting effect might have been behind the excellent performance initially observed on dataset A. Even though overfitting might have accounted for the differences in performance, we also observed a lot of noise in dataset B, even after the pre-processing stage. Some outliers were resilient to pre-processing because they could not be all completely removed without risking legitimate peaks ending up unrecognizable. These kinds of resilient outliers were also observed in dataset A. However, in that case, the resulting spikes were narrow, spaced wide apart, and scarce. Therefore, their contribution to the output of the moving median filter was insignificant. Conversely, resilient outliers in dataset B were abundant and very close to each other. Therefore, multiple nearby spikes managed to pass through the moving median filter in the form of a single spurious peak susceptible to being mistaken for a legitimate peak. On the other hand, the moving minimum filter in Algorithm-v2 was able to free the filtered signal from those spurious spikes and resulted in a remarkable improvement in performance compared to Algorithm-v1. Thus, we concluded that the poor performance of Algorithm-v1 was due to the moving median filter’s inability to cope with the noisy nature of the signals in dataset B.

The source of additional noise in dataset B was unclear. Functional decline (due to either normal aging or some condition, such as the frailty syndrome) imposes some mobility constraints on older people. Their movements become less precise and, during the experimentation, they might have accidentally moved the chair more prominently than their healthy young counterparts. For example, some of them needed to lean back and forth repeatedly to gain some momentum and stand up. However, we did not collect any data to support this hypothesis which, in fact, comes from an a posteriori subjective evaluation.

Algorithm-v2 was designed to be more responsive than Algorithm-v1 to spurious events in the signals. IRR results did indeed show a remarkable improvement. Again, the subset of signals in dataset B used in adjusting the algorithm parameters were also used in performance testing. Thus, an overfitting effect might have helped to reach better results than in the case of two separate sets. Even though overfitting might have accounted for such a good performance, we observed that the moving minimum filter in Algorithm-v2 was able to completely remove the effect of the spurious spikes from the filtered signal. Since we identified these spikes as responsible for the degradation in the performance of Algorithm-v1, we concluded that the moving minimum filter was responsible for the good performance of Algorithm-v2. Even though the noise from the resilient outliers and their corresponding spurious spikes were completely removed from the filtered signal, Algorithm-v2 and the manual count failed to show full correlation. We observed that the algorithm had difficulties in correctly spotting sit-to-stand transitions when the value of the minima in the filtered signal was greater than 30 cm. We think that this situation happened whenever an elder did not lean back after sitting down on the chair. However, we could not check this hypothesis because we did not video-record the experimental sessions.

The IRR for Algorithm-v2 on dataset B was good according to Koo and Li’s (2016) criteria because the ICC was over 0.75. However, since Algorithm-v2 and the manual count were not fully correlated, the error for some Algorithm-v2 scores was non-zero. We observed that the mean error for Algorithm-v2 scores nearby the border between the average class and the above-average class (m = 1.38, SD = 1.44) was similar or even greater than the mean distance between the manual count and that very same border (m = 1, SD = 1.22). Therefore, the relative magnitude of said error (138%) facilitated erroneous classifications of some subjects despite the good levels of ICC. Conversely, we observed that the mean error for Algorithm-v2 scores nearby the other border, i.e., between the below-average class the average class, (m = 0.94, SD = 1.18) was smaller than the mean distance between manual count and that very same border (m = 2.33, SD = 2.97). Therefore, below-average readings would be less prone to resulting in erroneous classifications. This is consistent with the fact that we only observed erroneous classifications between the average and the above-average classes. This effect resulted in a degradation of the classifier performance which, together with the low power of our sample, made it hard to tell whether outcomes from Algorithm-v2 are reliable enough to identify a subject’s functional status. In fact, the limited sample size was a general limitation of the study. The difference between 30-s CST scores measured two weeks apart is also used as a clinical criterion. It is used to track the progression of functional decline over time; however, our experimental design did not include any follow-up sessions. Thus, further experiments would be necessary to test the ability of Algorithm-v2 to generate scores consistent enough over time to raise the same red flags as a manual count would do.

We expected the main difficulty for the algorithms to be how to tell the difference between valid and invalid sit-to-stand transitions. This is because invalid transitions were expected to look like local maxima just like any other valid transition. Therefore, we expected erroneous outcomes to show a systematic overestimation of miscounts. However, miscounts did not seem to come from invalid transitions. No systematic overestimation was observed, and, in fact, invalid transitions were very scarce. From the total 335 sit-to-stand transitions in dataset B, only 11 of them were labeled as invalid, and they were scattered across seven different signals.

We studied the feasibility of using the quasi-periodic nature of the distance between a subject’s back and the chair backrest during a 30-s CST to carry out unsupervised measurements based on readings from a low-cost ultrasound sensor. The feasibility varied depending on the age and/or functional status of the user. The target population for this kind of clinical test involves older adults. Unfortunately, they generate such noisy signals that local maxima become hard to match to actual sit-to-stand transitions. Using a moving minimum filter to cancel the effects of said noise resulted in a remarkable improvement. Nevertheless, it is still not clear whether the results are good enough to make any clinical decisions based on the sensor outcomes. We did not find any other works reporting the processing of ultrasound readings for this purpose. Even though previous instrumented versions of the 30 s CST exist, all of them made use of acceleration and angular velocity signals from wearable IMUs. This latter approach was proven to be accurate, but the usability and the user experience of wearable devices for frail older people versus portable devices remain unexplored.

## Figures and Tables

**Table 1:** Participation criteria for older adults. Inclusion criteria (left) and exclusion criteria (right). CST—chair–stand test; SPPB—Short Physical Performance Battery.

A Subject could Enter the Study if ALL the Following Inclusion Criteria Applied.	A Subject could not Enter the Study if ANY of the following Exclusion Criteria Applied.
Subject is willing and able to give written informed consent for participation in the study. Subject is aged 70 years or older. Requirement to fulfill Fried’s criteria for frail or pre-frail individuals (FRIED ≥ 3 “frail”, FRIED = 0 “robust”) [4]. Patients able to perform the 30-s CST in a safe way. Patients able to perform SPPB in a safe way.	Subjects unwilling or unable to consent or unable to participate safely in intervention program. Clinically unstable patients in the clinical judgment of the investigator.

**Table 2:** Inter-rater reliability (IRR) results for Algorithm-v1. It performed very well on data from young healthy subjects (second column). However, performance dramatically dropped on data from older adults (third column). Not omitting unsuccessful sit-to-stand transitions did not improve Algorithm-v1 performance on data from older people (fourth column). ICC—intra-class correlation coefficient; CI—confidence interval.

	Dataset A: Algorithm-v1	Dataset B: Valid Events vs. Algorithm-v1	Dataset B: Total Events vs. Algorithm-v1
ICC (A,1)	0.96 Excellent	0.50 Moderate	0.50 Moderate
*p*-value	H1: ICC > 0.9 *p* = 0.03	H1: ICC > 1 *p* = 1 H1: ICC > 0.75 *p* = 0.99 H1: ICC > 0.5 *p* = 0.54	H1: ICC > 0.9 *p* = 1 H1: ICC > 0.75 *p* = 0.99 H1: ICC > 0.5 *p* = 0.50
95% CI	0.90, 0.98	0.15, 0.72	0.19, 0.73

**Table 3:** IRR results for Algorithm-v2. It performed well on data from older adults (second column). Not omitting unsuccessful sit-to-stand transitions did not improve Algorithm-v2 performance (third column).

	Dataset B: Valid Events vs. Algorithm-v2	Dataset B: Total Events vs. Algorithm-v2
ICC (A,1)	0.86 Moderate	0.89 Good
*p*-value	H1: ICC > 0.9 *p* = 0.82	H1: ICC > 0.9 *p* = 0.62
	H1: ICC > 0.75 *p* = 0.04	H1: ICC > 0.75 *p* = 0.01
	H1: ICC > 0.5 *p* = 0.00	H1: ICC > 0.5 *p* = 0.00
95% CI	0.73, 0.93	0.78, 0.95

**Table 4:** Results of the classifier performance assessment. Even though the accuracy was good (second row), not all values within the 95% CI (third row) were above the no information rate (NIR) (fourth row). However, the power of the statistical test was low (sixth row); thus, no firm conclusion could be stated.

	Dataset B, Algorithm-v2
Accuracy	0.86
95% CI	0.67, 0.96
NIR	0.75
*p*-value	H1: Accuracy > NIR *p* = 0.14
Power	Power = 0.42

## Supplementary Materials

The following are available online at https://www.mdpi.com/1424-8220/20/7/1975/s1.
